# RETRACTED: Sturtz, M.; House, C. Metal Catalysis Acting on Nitriles in Early Earth Hydrothermal Systems. *Life* 2023, *13*, 1524

**DOI:** 10.3390/life14040439

**Published:** 2024-03-26

**Authors:** Miranda Sturtz, Christopher House

**Affiliations:** Department of Geosciences, Pennsylvania State University, 116 Deike Building, University Park, PA 16802, USA

The *Life* Editorial Office retracts the article, “Metal Catalysis Acting on Nitriles in Early Earth Hydrothermal Systems” [1], cited above.

Following publication, the authors contacted the Editorial Office regarding the presentation of incorrect concentrations of compounds.

Adhering to our complaint’s procedure, an investigation was conducted by the Editorial Office that confirmed that, due to a calculation error, the concentrations of compounds used in the reactions were incorrectly reported in the millimolar (mM) range (e.g., 1 mM for each nitrile studied and 2 mM for sodium formate). Specifically, each NMR result and the reported visual changes to the experimental solutions due to polymerization are as shown in the manuscript, but these are only valid for molar concentrations (e.g., 1 M for each nitrile studied and 2 M for sodium formate). The observed polymerizations do not occur when the concentrations of nitriles are in the millimolar range. Given the scale of this miscalculation and the impact that this error has on the validity of the overall findings, the Editorial Office and Editorial Board have decided that a correction would not appropriately address this issue. Consequently, the Editorial Office, Editorial Board and authors have decided to retract this article [1] as per MDPI’s retraction policy (https://www.mdpi.com/ethics#_bookmark30) and in line with the Committee on Publication Ethics retraction guidelines (https://publicationethics.org/retraction-guidelines).

This retraction was approved by the Editor-in-Chief of the journal *Life*.

The authors agreed to this retraction.

## References

[1] Sturtz M., House C. (2023). RETRACTED: Metal Catalysis Acting on Nitriles in Early Earth Hydrothermal Systems. Life.

